# Three novel insect-associated species of *Simplicillium* (Cordycipitaceae, Hypocreales) from Southwest China

**DOI:** 10.3897/mycokeys.58.37176

**Published:** 2019-09-25

**Authors:** Wan-Hao Chen, Chang Liu, Yan-Feng Han, Jian-Dong Liang, Wei-Yi Tian, Zong-Qi Liang

**Affiliations:** 1 Department of Microbiology, Basic Medical School, Guizhou University of Traditional Chinese Medicine, Guiyang 550025, Guizhou, China; 2 School of Pharmacy, Guizhou University of Traditional Chinese Medicine, Guiyang 550025, Guizhou, China; 3 Institute of Fungus Resources, Department of Ecology, College of Life Sciences, Guizhou University, Guiyang 550025, Guizhou, China

**Keywords:** Commensal fungi, morphology, nutritional preference, phylogeny

## Abstract

In this paper, we introduce three new species of *Simplicillium*, viz. *S. cicadellidae*, *S. formicidae* and *S. lepidopterorum*, which were isolated from an infected leafhopper, ant and carpenterworm, respectively. Morphological comparisons and phylogenetic analyses based on multigene datasets (LSU+RPB1+RPB2+TEF and ITS+LSU) support the establishment of the three new species. *Simplicillium cicadellidae* was distinguished from other species in morphological characteristics by having smaller phialides and ellipsoidal conidia, and lacking octahedral crystals. The reverse of colonies were yellowish (#FFBF00), especially in the middle, and radially sulcate. *Simplicillium formicidae* was morphologically distinguished from other by having longer phialides and filiform to fusoid conidia, and by lacking octahedral crystals. *Simplicillium lepidopterorum* was morphologically distinguished from other species by having smaller, ellipsoidal to fusiform conidia, and by lacking octahedral crystals. The reverse of the colony was pale white. The three new species are likely to be nourished by plant to animal (especially insect) nutrients based on the evolutionary pattern of the Hypocreales, and they are described herein as being clearly distinct from other species in *Simplicillium*.

## Introduction

The genus *Simplicillium* W. Gams & Zare was introduced by Zare and Gams (2001) with *S. lanosoniveum* (J. F. H. Beyma) Zare & W. Gams as the type species. The genus is characterized with its complete lack of verticillate branching; mostly solitary phialides, which are discrete, aculeate and narrow and arise from aerial hyphae; conidia short-ellipsoidal to suglobose or obclavate, and adhering in globose heads or imbricate chains (Zare and Gams 2001). The members of *Simplicillium* are fungicolous and occur on various substrata (Zare and Gams 2001; Chen et al. 2008; Baiswar et al. 2014; Gauthier et al. 2014; Gomes et al. 2018). Furthermore, Zare and Gams (2001) introduced three additional species, viz., *S. lamellicola* (F. E. V. Sm.) Zare & W. Gams, *S. obclavatum* (W. Gams) Zare & W. Gams and *S. wallacei* H. C. Evans. The typical characteristics of *Simplicillium* include mostly solitary phialides, conidia adhering in globose, slimy heads or imbricate chains, and commonly present crystals in the agar (Zare and Gams 2001). Later, Zare and Gams (2008) transferred *S. wallacei* to *Lecanicillium* W. Gams & Zare based on the phylogenic analysis of internal transcribed spacer (ITS) region and this transfer was confirmed by Sung et al. (2007).

Liu and Cai (2012) reported a new species, *S. chinense* F. Liu & L. Cai, which was the first *Simplicillium* species from China. Five new *Simplicillium* species, *S. aogashimaense* Nonaka, Kaifuchi & Masuma, *S. cylindrosporum* Nonaka, Kaifuchi & Masuma, *S. minatense* Nonaka, Kaifuchi & Masuma, *S. subtropicum* Nonaka, Kaifuchi & Masuma and *S. sympodiophorum* Nonaka, Kaifuchi & Masuma were reported by Nonaka et al. (2013) from Tokyo, Japan. *Simplicillium calcicola* Z. F. Zhang, F. Liu & L. Cai, *S. coffeanum* A. A. M. Gomes & O. L. Pereira and *S. filiforme* R. M. F. Silva, R. J. V. Oliveira, Souza-Motta, J. L. Bezerra & G. A. Silva were reported by Zhang et al. (2017), Gomes et al. (2018) and Crous et al. (2018), respectively. Currently, *Simplicillium* consists of 12 species.

Kepler et al. (2017) re-evaluated the Cordycipitaceae based on the multigene dataset (SSU, LSU, TEF, RPB1 and RPB2), and indicated that *Simplicillium* species group in a clade and are the earliest diverging lineage in Cordycipitaceae. The nuclear ribosomal ITS and LSU were first used to identify cryptic diversification among *Simplicillium* species by Liu and Cai (2012) and then were widely applied in the identification of *Simplicillium* species by Nonaka et al. (2013), Zhang et al. (2017), Gomes et al. (2018) and Crous et al. (2018).

Zare and Gams (2001) noted that *Simplicillium* species were found on various substrata and fungi. Other substrata were found later, such as limstone and wood (Liu and Cai 2012; Zhang et al. 2017). Many bioactive compounds were discovered in *Simplicillium*, such as alkaloids (Fukuda et al. 2014), peptides (Liang et al. 2016; 2017; Dai et al. 2018), diketopiperazine (Yan et al. 2015), xylanases (Roy et al. 2013), anthraquinones (Huang et al. 2015), antibiotics (Takata et al. 2013; Dong et al. 2018), and especially Simpotentin, which is a new potentiator of amphotericin B activity against *Candida albicans* (C. P. Robin) Berkhout and has showed great potential applications in medicine (Uchida et al. 2019). Furthermore, the antimicrobial activities and entomopathogenicity has meant that *Simplicillium* has potential applications in biocontrol (Ward et al. 2012; Zhao et al. 2013; Le Dang et al. 2014; Lim et al. 2014; Chen et al. 2017; Skaptsov et al. 2017). However, as far as we know, there are limited reports of *Simplicillium* species isolated from infected insects.

Three infected insect specimens were found during a survey of araneogenous fungi and allies from southwestern China. Some fungal strains were isolated and purified from the three specimens. Based on polyphasic approach (morphological, ecological characteristics along with a phylogenetic analysis), they were identified as three new species, *Simplicillium cicadellidae* sp. nov., *S. formicidae* sp. nov. and *S. lepidopterorum* sp. nov.

## Materials and methods

### Collection and isolation

Three infected insect specimens (DL1004, GY1101 and GY2913) were collected from Dali, Rongjiang Country (26°01'58.70"N, 108°24'48.06"E) and Tongmuling (26°23'25.92"N, 106°41'3.35"E), Huaxi District, Guizhou Province, on 1 October, 9 November and 31 July, 2018, respectively. The surface of the specimens were rinsed with sterile water, followed by surface sterilization with 75% ethanol for 3–5 s. A part of the insect body was cut off and used to inoculate a piece of tissue in haemocoel on potato dextrose agar (PDA) and improved potato dextrose agar (PDA, 1% w/v peptone) (Qu et al. 2018). The strain was isolated and cultured at 22 °C for 14 d under 12 h light/12 h dark conditions following protocols described by Zou et al. (2010). Strains DL10041, DL10042, GY11011, GY11012, GY29131 and GY29132 were obtained.

### Culture and identification

The strains were incubated in PDA at 25 °C for 14 d. Macroscopic and microscopic morphological characteristics of the fungi were examined using classical mycological techniques, and the growth rates were determined. The fresh hyphae were observed with an optical microscope (OM, BX35, Olympus, Japan) following pretreatment with lactophenol cotton blue solution or normal saline. The ex-type cultures and dried culture as holotype specimens were deposited in GZAC, Guizhou University, Guiyang, China.

### DNA extraction, PCR amplification and nucleotide sequencing

DNA extraction was carried out in accordance with Liang et al. (2009). The extracted DNA was stored at −20 °C. The amplification of large subunit ribosomal RNA (LSU) genes was performed using NS1-1/AB28 primers (Curran et al. 1994). Translation elongation factor 1 alpha (TEF) and DNA-directed RNA polymerase II largest subunit 2 (RPB2) were amplified using 983F/2218R and RPB2-5F/RPB2-7Cr primers according to van den Brink et al. (2012). DNA-directed RNA polymerase II largest subunit 1 (RPB1) was amplified with the primer pair CRPB1 and RPB1-Cr (Castlebury et al. 2004). The internal transcribed spacer (ITS) region was amplified using ITS4/ITS5 primers by PCR following the procedures described by White et al. (1990). PCR products were purified using the UNIQ-10 column PCR products purification kit [no. SK1141; Sangon Biotech (Shanghai) Co., Shanghai, China] in accordance with the manufacturer’s protocol and sequenced at Sangon Biotech (Shanghai) Co. The resulting sequences were submitted to GenBank.

The new species *Simplicillium cicadellidae*, *S. formicidae* and *S. lepidopterorum* were registered in MycoBank with the numbers MB 831336, MB 831337 and MB 831335, respectively.

### Sequence alignment and phylogenetic analyses

DNA sequences generated in this study were assembled and edited using DNASTAR Lasergene software (version 6.0). Sequences of ITS, LSU, RPB1, RPB2 and TEF were selected based on previously published data by Nonaka et al. (2013), Zhang et al. (2017), Gomes et al. (2018), Crous et al. (2018) and Mongkolsamrit et al. (2018). Multiple sequence alignments for ITS, LSU, RPB1, RPB2 and TEF were carried out using MAFFT v7.037b (Katoh and Standley 2013). Sequence editing was performed with MEGA6 (Tamura et al. 2013), and the resulting output was in Fasta file format. The concatenated LSU+RPB1+RPB2+TEF and ITS+LSU sequences were assembled by SequenceMatrix v.1.7.8 (Vaidya et al. 2011). Gene concordance was assessed with the ‘hompart’ command in PAUP4.0b10 (Swofford 2002).

Two different analyses have been carried out using Bayesian inference (BI) and maximum likelihood (ML) methods. Analysis 1: To check the relationship between *Simplicillium* species and its allies in Cordycipitaceae based on the combined dataset of (LSU+RPB1+RPB2+TEF). Analysis 2: To check the relationship among *Simplicillium* spp. based on the combined dataset of (ITS+LSU). For the BI analysis, two runs were executed simultaneously for 10,000,000 generations, saving trees every 500 generations, with the GTR+G nucleotide substitution model across all the partitions, in MrBayes 3.2 (Ronquist et al. 2012). After the analysis was finished, each run was examined with the program Tracer v1.5 (Drummond and Rambaut 2007) to determine burn-in and confirm that both runs had converged. For the ML analysis in RAxML (Stamatakis 2014), the GTRGAMMA model was used for all the partitions in accordance with recommendations in the RAxML manual against the use of invariant sites. The final alignment is available from TreeBASE under submission ID: 24549 (http://www.treebase.org)

## Results

### Phylogenetic analyses

**Figure 1.**
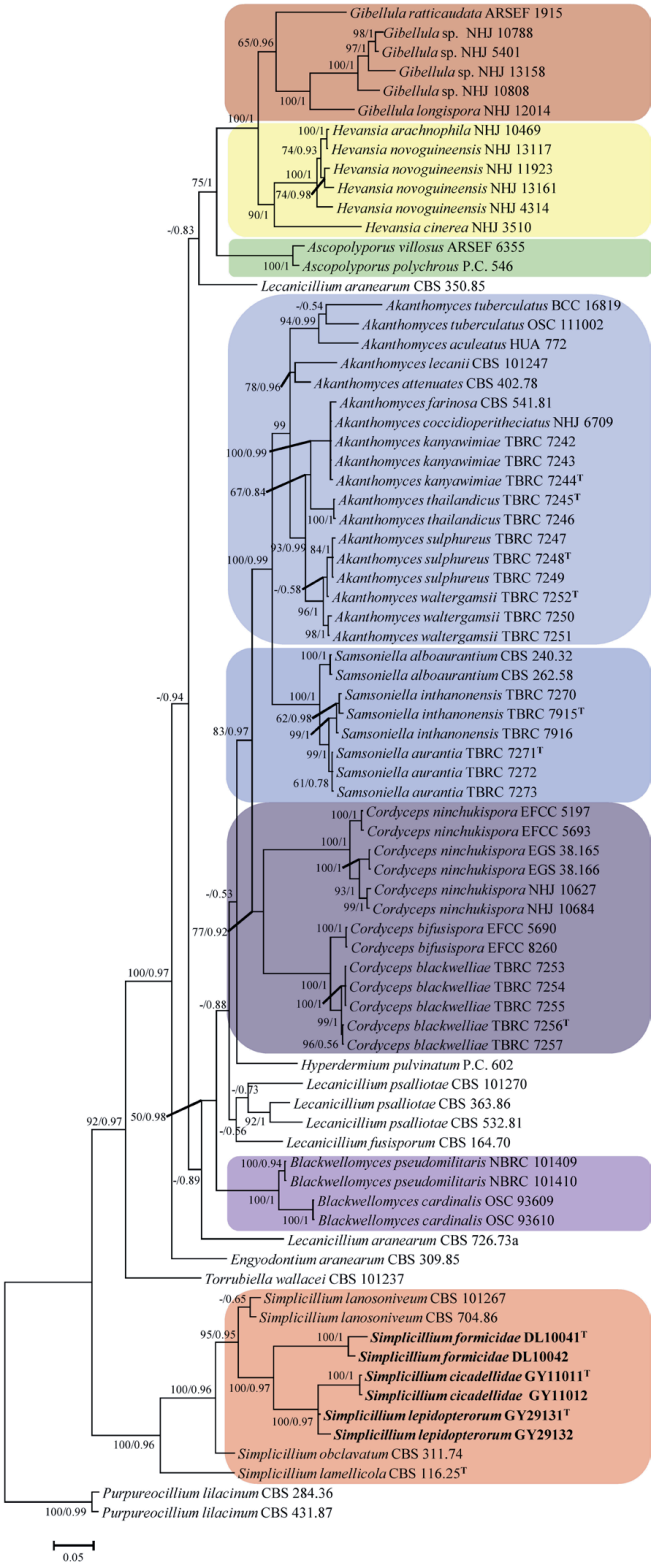
Phylogenetic relationships among the genus *Simplicillium* and its allies in Cordycipitaceae based on multigene dataset (LSU, RPB1, RPB2 and TEF). Statistical support values (≥ 0.5/50%) are shown at the nodes for ML bootstrap support/BI posterior probabilities. The tree is rooted with *Purpureocillium lilacinum* (CBS 284.36 and CBS 431.87). The new species are in bold face. T in the upper right corner indicates the type strains.

A phylogenetic tree of *Simplicillium* in Cordycipitaceae was generated from the maximum-likelihood (ML) and Bayesian inference (BI) based on a combined data set of LSU, RPB1, RPB2 and TEF sequence data. Statistical support (≥ 50%/0.5) is shown at the nodes for ML bootstrap support/BI posterior probabilities (Fig. 1). The strain numbers are noted after each species’ name. The tree is rooted with *Purpureocillium lilacinum* (Thom) Luangsa-ard, Houbraken, Hywel-Jones & Samson (CBS 284.36 and CBS 431.87). The concatenated sequences including 40 taxa and contained 2,205 characters with gaps (LSU: 447, RPB1: 518, RPB2: 560, and TEF: 680).

A phylogenetic tree of *Simplicillium* species level was generated from the maximum-likelihood (ML) and Bayesian inference (BI) analysis based on a combined data set of ITS and LSU sequence data set. Statistical support (≥ 50%/0.5) are shown at the nodes for ML bootstrap support/BI posterior probabilities. The strain numbers are noted after each species’ name. The tree is rooted with *Pochonia chlamydosporia* (Goddard) Zare & W. Gams (CBS 103.65). The dataset includes 16 taxa and consists of 1,000 characters with gaps (ITS: 489 and LSU: 511).

Analysis 1: family Cordycipitaceae. The RAxML analysis of the combined dataset (LSU+RPB1+RPB2+TEF) yielded a best scoring tree (Fig. 1) with a final ML optimization likelihood value of –24,337.973328. Parameters for the GTR model of the concatenated data set was as follows: estimated base frequencies; A = 0.242689, C = 0.276532, G = 0.270879, T = 0.209901; substitution rates AC = 0.926706, AG = 2.728719, AT = 0.823168, CG = 0.803225, CT = 6.257555, GT = 1.000000; gamma distribution shape parameter α = 0.410435. The Bayesian analysis resulted in 20,001 trees after 10,000,000 generations. The first 4,000 trees, representing the burn-in phase of the analyses, were discarded, while the remaining 16,001 trees were used for calculating posterior probabilities in the majority rule consensus tree. In the phylogenetic tree (Fig. 1), *Simplicillium cicadellidae*, *S. formicidae* and *S. lepidopterorum* cluster with other *Simplicillium* species in a clade, and within the earliest diverging lineage in Cordycipitaceae.

**Figure 2.**
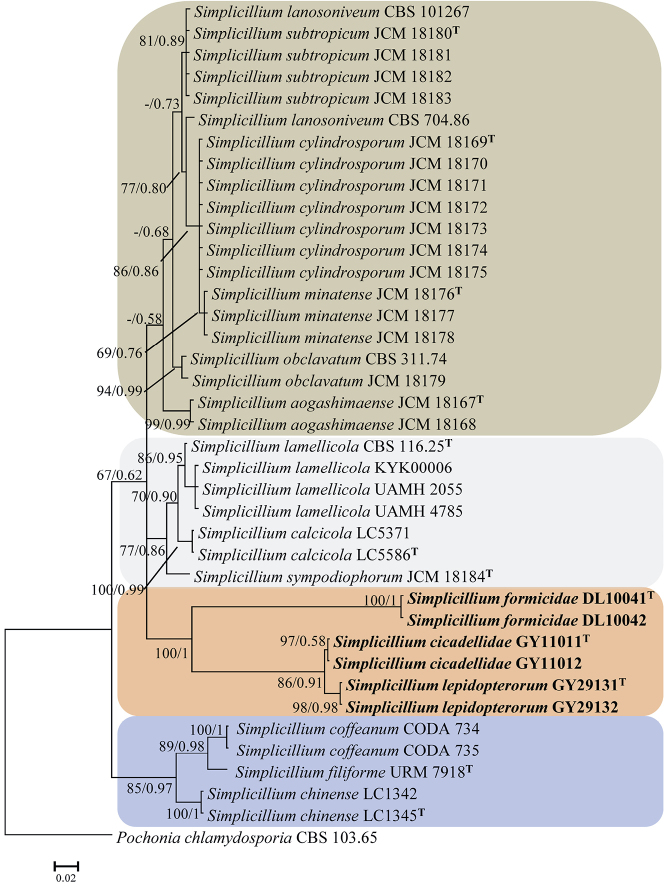
Phylogenetic relationships among the new taxa *S. cicadellidae*, *S. formicidae*, *S. lepidopterorum* and other *Simplicillium* species by ITS+LSU sequences. Statistical support values (≥ 0.5/50%) are shown at the nodes for ML bootstrap support/BI posterior probabilities. The tree is rooted with *Pochonia chlamydosporia* (CBS 103.65). The new species are in bold face. T in the upper right corner indicates the type strains.

Analysis 2: *Simplicillium* species. The RAxML analysis of the combined dataset (ITS+LSU) yielded a best scoring tree (Fig. 2) with a final ML optimization likelihood value of –4,849.039588. Parameters for the GTR model of the concatenated data set was as follows: Estimated base frequencies; A = 0.243952, C = 0.258870, G = 0.268223, T = 0.228956; substitution rates AC = 1.296760, AG = 2.678402, AT = 1.354112, CG = 1.488619, CT = 5.097242, GT = 1.000000; gamma distribution shape parameter α = 0.462419. The Bayesian analysis resulted in 20,001 trees after 10,000,000 generations. The first 4,000 trees, representing the burn-in phase of the analyses, were discarded, while the remaining 16,001 trees were used for calculating posterior probabilities in the majority rule consensus tree. In the phylogenetic tree (Fig. 2), *Simplicillium* species were resolved into four obvious clades. *S. cicadellidae*, *S. formicidae* and *S. lepidopterorum* were nested in a subclade and formed three independent branches, which received maximum statistical support (BI posterior probabilities 1, ML bootsrap 100%).

**Table 1.** Taxa included in the phylogenetic analyses

Species	Strain No.	GenBank Accession No.				
		ITS	LSU	RPB1	RPB2	TEF
*Akanthomyces aculeatus*	HUA 772		KC519370			KC519366
*A. attenuates*	CBS 402.78		AF339565	EF468888	EF468935	EF468782
*A. coccidioperitheciatus*	NHJ 6709		EU369042	EU369067	EU369086	EU369025
*A. farinosa*	CBS 541.81					JQ425686
*A. kanyawimiae*	TBRC 7242		MF140718	MF140784	MF140808	MF140838
	TBRC 7243		MF140717	MF140783	MF140807	MF140837
	TBRC 7244		MF140716			MF140836
*A. lecanii*	CBS 101247		AF339555	DQ522407	DQ522466	DQ522359
*A. sulphureus*	TBRC 7247		MF140720			MF140841
	TBRC 7248		MF140722	MF140787	MF140812	MF140843
	TBRC 7249		MF140721	MF140786	MF140734	MF140842
*A. thailandicus*	TBRC 7245				MF140809	MF140839
	TBRC 7246		MF140719		MF140810	MF140840
*A. tuberculatus*	BCC 16819		GQ249987			GQ250037
	OSC 111002		DQ518767	DQ522384	DQ522435	DQ522338
*A. waltergamsii*	TBRC 7250		MF140715			MF140835
	TBRC 7251		MF140713	MF140781	MF140805	MF140833
	TBRC 7252		MF140714	MF140782	MF140806	MF140834
*Ascopolyporus polychrous*	P.C. 546		DQ118737	DQ127236		DQ118745
*A. villosus*	ARSEF 6355		AY886544	DQ127241		DQ118750
*Blackwellomyces cardinalis*	OSC 93609		AY184962	DQ522370	DQ522422	DQ522325
	OSC 93610		AY184963	EF469088	EF469106	EF469059
*B. pseudomilitaris*	NBRC 101409		JN941393	JN992482		
	NBRC 101410		JN941394	JN992481		
*Cordyceps bifusispora*	EFCC 5690		EF468806	EF468854	EF468909	EF468746
	EFCC 8260		EF468807	EF468855	EF468910	EF468747
*C. blackwelliae*	TBRC 7253		MF140705	MF140774	MF140798	MF140825
	TBRC 7254		MF140704	MF140773	MF140797	MF140824
	TBRC 7255		MF140703	MF140772	MF140796	MF140823
	TBRC 7256		MF140702	MF140771	MF140795	MF140822
	TBRC 7257		MF140701	MF140770	MF140794	MF140821
*C. ninchukispora*	EFCC 5197		EF468820	EF468868		EF468760
	EFCC 5693		EF468821	EF468869		EF468762
	EGS 38.165		EF468846	EF468900		EF468795
	EGS 38.166		EF468847	EF468901		EF468794
	NHJ 10627		EF468822	EF468870		EF468763
	NHJ 10684		EF468823	EF468871		EF468761
*Engyodontium aranearum*	CBS 309.85		AF339526	DQ522387	DQ522439	DQ522341
*Gibellula longispora*	NHJ 12014			EU369055	EU369075	EU369017
*G. pulchra*	NHJ 10808		EU369035	EU369056	EU369076	EU369018
*G. ratticaudata*	ARSEF 1915		DQ518777	DQ522408	DQ522467	DQ522360
*Gibellula* sp.	NHJ 5401			EU369059	EU369079	
	NHJ 10788		EU369036	EU369058	EU369078	EU369019
	NHJ 13158		EU369037	EU369057	EU369077	EU369020
*Hevansia arachnophila*	NHJ 10469		EU369031	EU369047		EU369008
*H. cinerea*	NHJ 3510			EU369048	EU369070	EU369009
*H. novoguineensis*	NHJ 4314			EU369051	EU369071	EU369012
	NHJ 11923		EU369032	EU369052	EU369072	EU369013
	NHJ 13117			EU369049	EU369073	EU369010
	NHJ 13161			EU369050		EU369011
*Hyperdermium pulvinatum*	P.C. 602		AF242353	DQ127237		DQ118746
*L. aranearum*	CBS 726.73a		AF339537	EF468887	EF468934	EF468781
*L. fusisporum*	CBS 164.70**T**		AF339549	EF468889		EF468783
*L. psalliotae*	CBS 363.86**T**		AF339559	EF468890		EF468784
	CBS 532.81		AF339560	EF469096	EF469112	EF469067
	CBS 101270		EF469081	EF469095	EF469113	EF469066
*Pochonia chlamydosporia*	CBS 103.65	MH858504				
*Purpureocillium lilacinum*	CBS 284.36		FR775484	EF468898	EF468941	EF468792
	CBS 431.87		EF468844	EF468897	EF468940	EF468791
*Samsoniella alboaurantium*	CBS 240.32		JF415979	JN049895	JF415999	JF416019
	CBS 262.58		MG665232			JQ425685
*S. aurantia*	TBRC 7271**T**		MF140728	MF140791	MF140818	MF140846
	TBRC 7272		MF140727	MF140817		MF140845
	TBRC 7273		MF140726		MF140816	MF140844
*S. inthanonensis*	TBRC 7915**T**		MF140725	MF140790	MF140815	MF140849
	TBRC 7916		MF140724	MF140789	MF140814	MF140848
	TBRC 7270		MF140723	MF140788	MF140813	MF140847
*Simplicillium aogashimaense*	JCM 18167**T**	AB604002				
	JCM 18168	AB604004				
*S. calcicola*	LC 5371	KU746705	KU74675			
	LC 5586**T**	KU746706	KU746752			
*S. chinense*	LC 1342	JQ410323	JQ410321			
	LC 1345	NR155782	JQ410322			
***S. cicadellidae***	**GY11011T**	MN006243	MN006249	MN022271		MN022263
	**GY11012**	MN006244	MN006250	MN022272		MN022264
*S. coffeanum*	COAD 2057**T**	MF066034	MF066032			
	COAD 2061	MF066035	MF066033			
*S. cylindrosporum*	JCM 18169**T**	AB603989				
	JCM 18170	AB603994				
	JCM 18171	AB603997				
	JCM 18172	AB603998				
	JCM 18173	AB603999				
	JCM 18174	AB604005				
	JCM 18175	AB604006				
*S. filiforme*	URM 7918	MH979338	MH979399			
***S. formicidae***	**DL10041T**	MN006241	MN006247	MN022269	MN022267	
	**DL10042**	MN006242	MN006248	MN022270	MN022268	
*S. lamellicola*	CBS 116.25**T**	AJ292393	AF339552	DQ522404	DQ522462	DQ522356
	UAMH 2055	AF108471				
	UAMH 4785	AF108480				
*S. lamellicola* ^b^	KYK00006	AB378533				
*S. lanosoniveum*	CBS 704.86	AJ292396	AF339553	DQ522406	DQ522464	DQ522358
	CBS 101267	AJ292395	AF339554	DQ522405	DQ522463	DQ522357
***S. lepidopterorum***	**GY29131T**	MN006246	MN006251	MN022273		MN022265
	**GY29132**	MN006245	MN006252	MN022274		MN022266
*S. minatense*	JCM 18176**T**	AB603992				
	JCM 18177	AB603991				
	JCM 18178	AB603993				
*S. obclavatum*	CBS 311.74**T**	AJ292394	AF339517			EF468798
	JCM 18179	AB604000				
*S. subtropicum*	JCM 18180**T**	AB603990				
	JCM 18181	AB603995				
	JCM 18182	AB603996				
	JCM 18183	AB604001				
*S. sympodiophorum*	JCM 18184**T**	AB604003				
*Torrubiella wallacei*	CBS 101237**T**		AY184967	EF469102	EF469119	EF469073

**T**= type strains, strain and sequences generated in this study are shown in bold.

## Taxonomy

### Simplicillium cicadellidae

Taxon classificationFungiHypocrealesCordycipitaceae

W.H. Chen, C. Liu, Y.F. Han, J.D. Liang, Z.Q. Liang sp. nov.

62370442-F973-5FBB-B628-8AFF09A70BB1

831336

Figure 3

#### Etymology.

The epithet *cicadellidae* refers to an insect host in family Cicadellidea.

#### Diagnosis.

Characterized by phialides always solitary and rather long and narrow, 12.9–18.3 × 0.8–1.1 μm. Conidia adhering in globose slimy heads, mostly ellipsoidal, 1.8–2.8 × 1.4–1.8 μm. Octahedral crystals absent. Reverse of colony yellowish, especially in the middle, and radially sulcate.

#### Type.

CHINA, Guizhou Province, Huaxi District (26°23'25.92"N, 106°41'3.35"E), 9 November 2018, Wanhao Chen, **holotype**GZAC GY1101, ex-type culture GZAC GY11011. Sequences from isolated strain GY11011 has been deposited in GenBank with accession numbers: ITS = MN006243, LSU = MN006249, RPB1 = MN022271 and TEF = MN022263.

**Figure 3.**
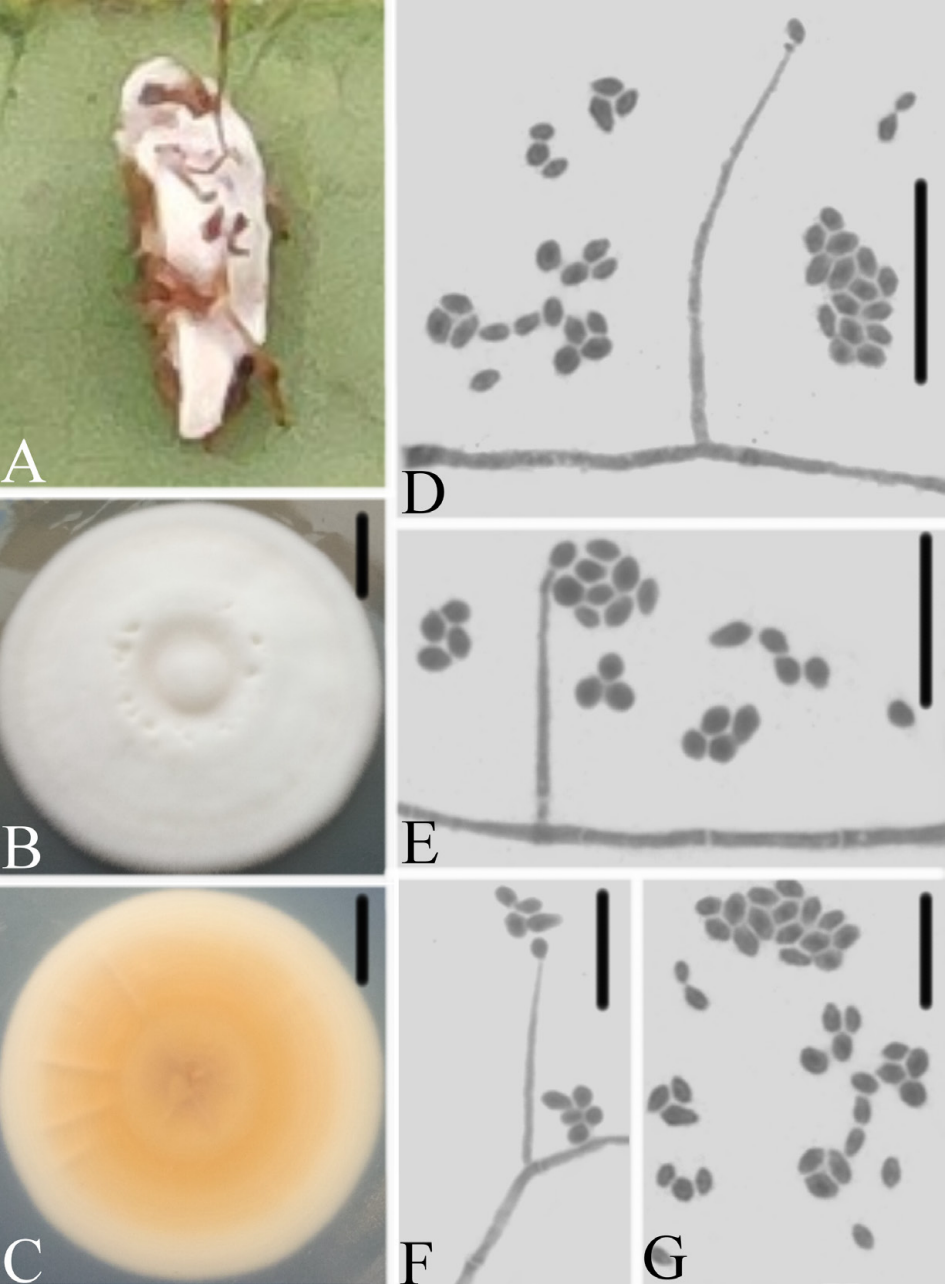
*Simplicillium cicadellidae***A** infected leafhopper (Hemiptera) **B–C** culture plate, showing the front (**B**) and the reverse (**C**) of the colony, cultured on PDA medium **D–F** phialides solitary, conidia adhering ellipsoidal slimy head and conidia **G** conidia. Scale bars: 10 mm (**B, C**), 10μm (**D, E, F, G**).

#### Description.

Colonies reaching 45–47 mm in diameter in 14 d on PDA; white; reverse yellowish, especially in the middle, and radially sulcate. Hyphae septate, hyaline, smooth-walled, 0.9–1.9 μm wide. Phialides arising from aerial hyphae, gradually tapering towards apex, without basal septa, always solitary and rather long and narrow, 12.9–18.3 × 0.8–1.1 μm. Conidia adhering in ellipsoidal slimy heads, mostly ellipsoidal, hyaline, smooth-walled, 1.8–2.8 × 1.4–1.8 μm. Octahedral crystals absent.

#### Host.

Leafhopper (Hemiptera)

#### Distribution.

Huaxi District, Guizhou Province, China

#### Remarks.

Zare and Gams (2001) summarized the typical characteristics of *Simplicillium* as having mostly solitary phialides arising from aerial hyphae, conidia adhering in globose slimy heads or imbricate chains, crystals commonly present, fungicolous and on various other substrata. *Simplicillium cicadellidae* was easily identified as belonging to *Simplicillium* because of its solitary phialides, conidia adhering in ellipsoidal slimy heads, and lack of octahedral crystals. Comparing with the typical characteristics of 12 species (Table 2), it was easily distinguished from other species in having the phialides always solitary and rather long and narrow (12.9–18.3 × 0.8–1.1 μm), the conidia adhering in globose slimy heads, which are mostly ellipsoidal (1.8–2.8 × 1.4–1.8 μm), and the octahedral crystals absent. The reverse of colony was yellowish, especially in the middle, and radially sulcate. Based on ITS and LSU rDNA, *S. cicadellidae* is phylogenetically close to *S. formicidae* and *S. lepidopterorum*. However, *S. cicadellidae* has ellipsoidal conidia and shorter phialides (12.9–18.3 × 0.8–1.1 μm), and the reverse of colony was yellowish.

**Table 2.** Morphological comparison of three new species with other *Simplicillium* species

**Species**	**Morphological characteristics**				**Notes**
	**Phialide (Conidiogenous cell) (μm)**	**Conidia (μm)**	**Conidia mass**	**Octahedral crystals**	
*S. aogashimaense* ^a^	(19–)23–53 × 1.2–2.0	cylindrical, 4.2–6.5 × 1.2–2.0	globose heads	present	Chlamydospores present
*S. calcicola* ^b^	14–38 × 1–2	micro-: globose, oval or ellipsoidal, 2–3.5 × 1–1.5		absent	
		macro-: fusiform, 4.5–8 × 1–2			
*S. chinense* ^c^	(6.0–)15–30(–68.0) × 1.5	oval, ellipsoidal or cylindrical 3.5–5.0 × 1.0–1.5	branched or unbranched chains	present	
*S. coffeanum* ^d^	11–40(–70) × 1.0–2.4	micro-: spindle-shaped, 5.3–8.8 × 1.0–1.6	subglobose to ellipsoidal heads	absent	
		macro-: ellipsoidal to fusiform, 2.2–3.8 × 0.8–1.5			
*S. cylindrosporum* ^a^	17–32 × 1.2–2.0(–2.5)	cylindrical, 3.0–4.5(–5.0) × 1.0–2.0	globose heads	present	
*S. filiforme* ^e^	9–18 × 1	fusoid to filiform, 7.2–12.5 × 1	zigzag chains	absent	
*S. lamellicola* ^f^	15–50 × 0.7–1.0	micro-: spindle-shaped, 4.5–9.0 × 0.8–1.2	subglobose to ellipsoidal heads	present	
		macro-: oval to ellipsoidal, 2.0–3.0 × 0.7–1.2			
*S. lanosoniveum* ^f^	15–35 × 0.7–1.5	subglobose, oval, ellipsoidal 1.5–3 × 0.7–1.3	globose heads	present	
*S. minatense* ^a^	11–31(–47) × 1.0–1.7	globose to subglobose, sometimes ellipsoidal, 2.0–3.5 × 1.8–2.5(–2.8)	globose heads	present	
*S. obclavatum* ^f^	30–52 × 0.8–1.2	obclavate to ellipsoidal, 2.5–3.5 × 1–2	short imbricate chains	present	
*S. subtropicum* ^a^	(15–)20–42(–50) × 1.0–2.3	subglobose to ellipsoidal, 2.3–4.0(–4.5) × 1.5–3.3	globose heads	present	
*S. sympodiophorum* ^a^	20–34(–47) × 0.5–1.3 denticles present	oval to ellipsoidal, 2.2–3.5 × 1.0–2.0		present	
*S. cicadellidae*	12.9–18.3 × 0.8–1.1	ellipsoidal, 1.8–2.8 × 1.4–1.8	ellipsoidal heads	absent	colonies reverse pale white
*S. formicidae*	51–70.1 × 0.7–0.9	filiform to fusoid, 3.9–7.9 × 0.8–1.3	globose heads	absent	
*S. lepidopterorum*	15.3–26.2 × 0.7–1.4	ellipsoidal, 1.6–2.4 × 1.4–1.7	globose heads	absent	colonies reverse yellowish

a–f: data are derived from Zare and Gams (2001), Nonaka et al. (2013), Zhang et al. (2017), Liu and Cai 2012, Gomes et al. (2018) and Crous et al. (2018), respectively.

### Simplicillium formicidae

Taxon classificationFungiHypocrealesCordycipitaceae

W.H. Chen, C. Liu, Y.F. Han, J.D. Liang, Z.Q. Liang sp. nov.

36C3EC82-58AB-5D06-A977-243E3CF2DBEB

831337

Figure 4

#### Etymology.

The epithet *formicidae* refers to an insect host in family Formicidae.

#### Diagnosis.

Characterized by phialides always being solitary and rather long and narrow, 51–70.1 × 0.7–0.9 μm. Conidia adhering in globose slimy heads, mostly filiform to fusoid, 3.9–7.9 × 0.8–1.3 μm. Octahedral crystals absent.

#### Type.

CHINA, Guizhou Province, Rongjiang County (26°01'58.70"N, 108°24'48.06"E), 1 October 2018, Wanhao Chen, **holotype**GZAC DL1004, ex-type culture GZAC DL10041. Sequences from isolated strain DL10041 has been deposited in GenBank with accession numbers: ITS = MN006241, LSU = MN006247, RPB1 = MN022269 and RPB2 = MN022267.

**Figure 4.**
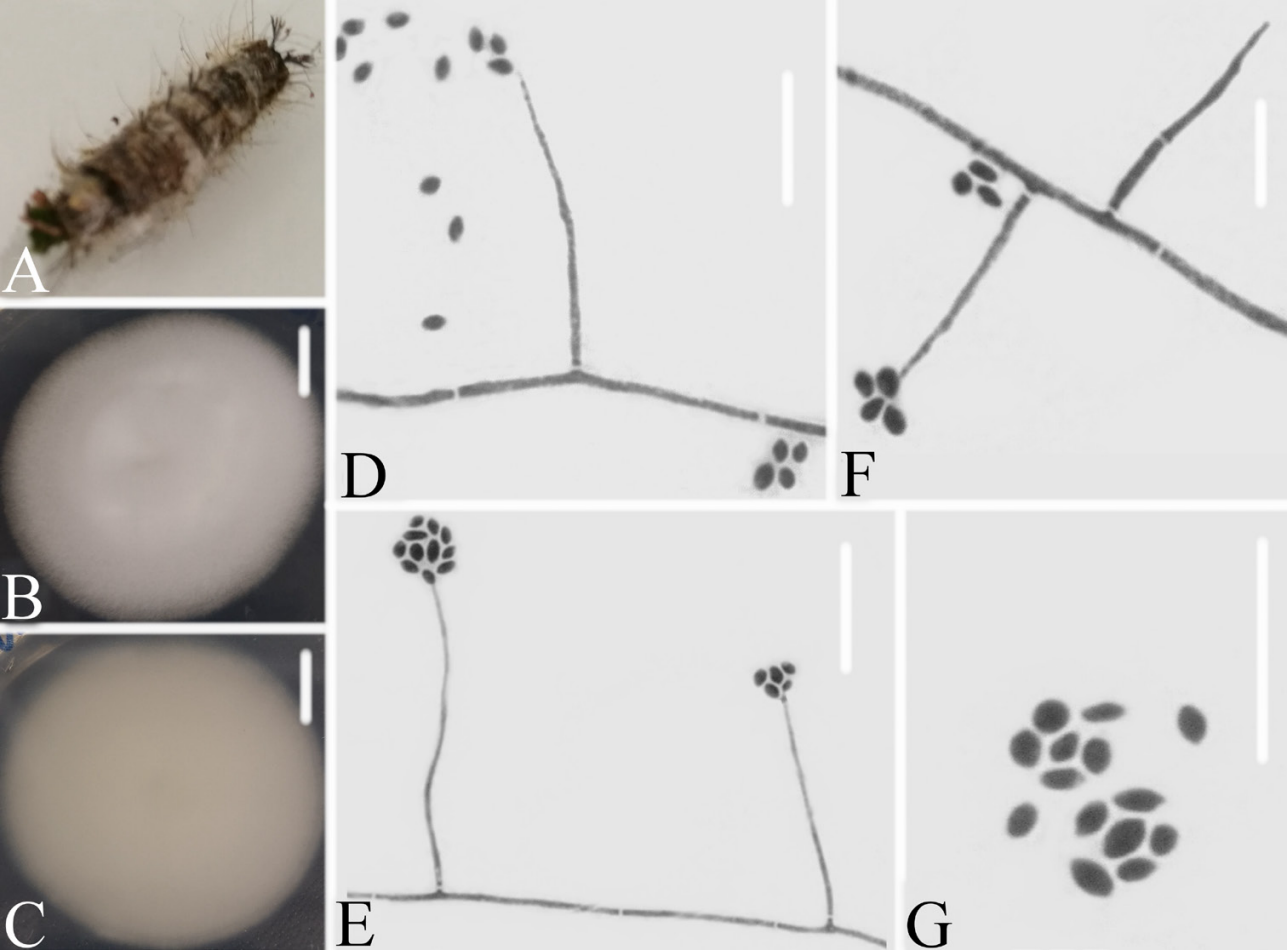
*Simplicillium lepidopterorum***A** infected carpenterworm (Lepidoptera) **B, C** culture plate, showing the front (**B**) and the reverse (**C**) of the colony, cultured on PDA medium **D, E, F** phialides solitary and conidia in globose heads **D** conidia. Scale bars: 10 mm (**B, C**), 10μm (**D, E, F, G**).

#### Description.

Colonies reaching 26–32 mm in diameter in 14 d on PDA; white; reverse pale brown to brown, and with brown secretions. Hyphae septate, hyaline, smooth-walled, 1.2–1.8 μm wide. Phialides arising from aerial hyphae, gradually tapering towards the apex, without basal septa, always solitary and rather long and narrow, 51–70.1 × 0.7–0.9 μm. Conidia adhering in globose slimy heads, mostly filiform to fusoid, hyaline, smooth-walled, 3.9–7.9 × 0.8–1.3 μm. Octahedral crystals absent.

#### Host.

Ant (Hymenoptera)

#### Distribution.

Rongjiang County, Guizhou Province, China

#### Remarks.

*Simplicillium formicidae* was easily identified as belonging to *Simplicillium* because of its solitary phialides, conidia adhering in globose slimy heads, and lack of octahedral crystals. Compared with the typical characteristics of 12 species (Table 2), it was easily distinguished from those species by having the phialides always solitary and rather long and narrow (51–70.1 × 0.7–0.9 μm) and the conidia mostly filiform to fusoid (3.9–7.9 × 0.8–1.3 μm), and adhering in globose slimy heads, and in having octahedral crystals absent. Based on ITS and LSU rDNA, *S. formicidae* is phylogenetically close to *S. cicadellidae* and *S. lepidopterorum*. However, *S. formicidae* has larger filiform to fusoid conidia (3.9–7.9 × 0.8–1.3 μm).

### Simplicillium lepidopterorum

Taxon classificationFungiHypocrealesCordycipitaceae

W.H. Chen, C. Liu, Y.F. Han, J.D. Liang & Z.Q. Liang sp. nov.

E1655909-20BA-5B52-B1F5-014F2D373CA9

831335

Figure 5

#### Etymology.

The epithet *lepidopterorum* refers to an insect host in order Lepidoptera.

#### Diagnosis.

Characterized by phialides always being solitary and rather long and narrow, 15.3–26.2 × 0.7–1.4 μm, Conidia adhering in globose slimy heads, mostly ellipsoidal, 1.6–2.4 × 1.4–1.7 μm. Octahedral crystals absent. The reverse of colony was pale white.

#### Type.

CHINA, Guizhou Province, Huaxi District (26°23'25.92"N, 106°41'3.35"E), 31 July 2018, Wanhao Chen, **holotype**GZAC GY2913, ex-type culture GZAC GY29131, sequences from isolated strain GY29131 has been deposited in GenBank with accession numbers: ITS = MN006246, LSU = MN006251, RPB1 = MN022273 and TEF = MN022265.

**Figure 5.**
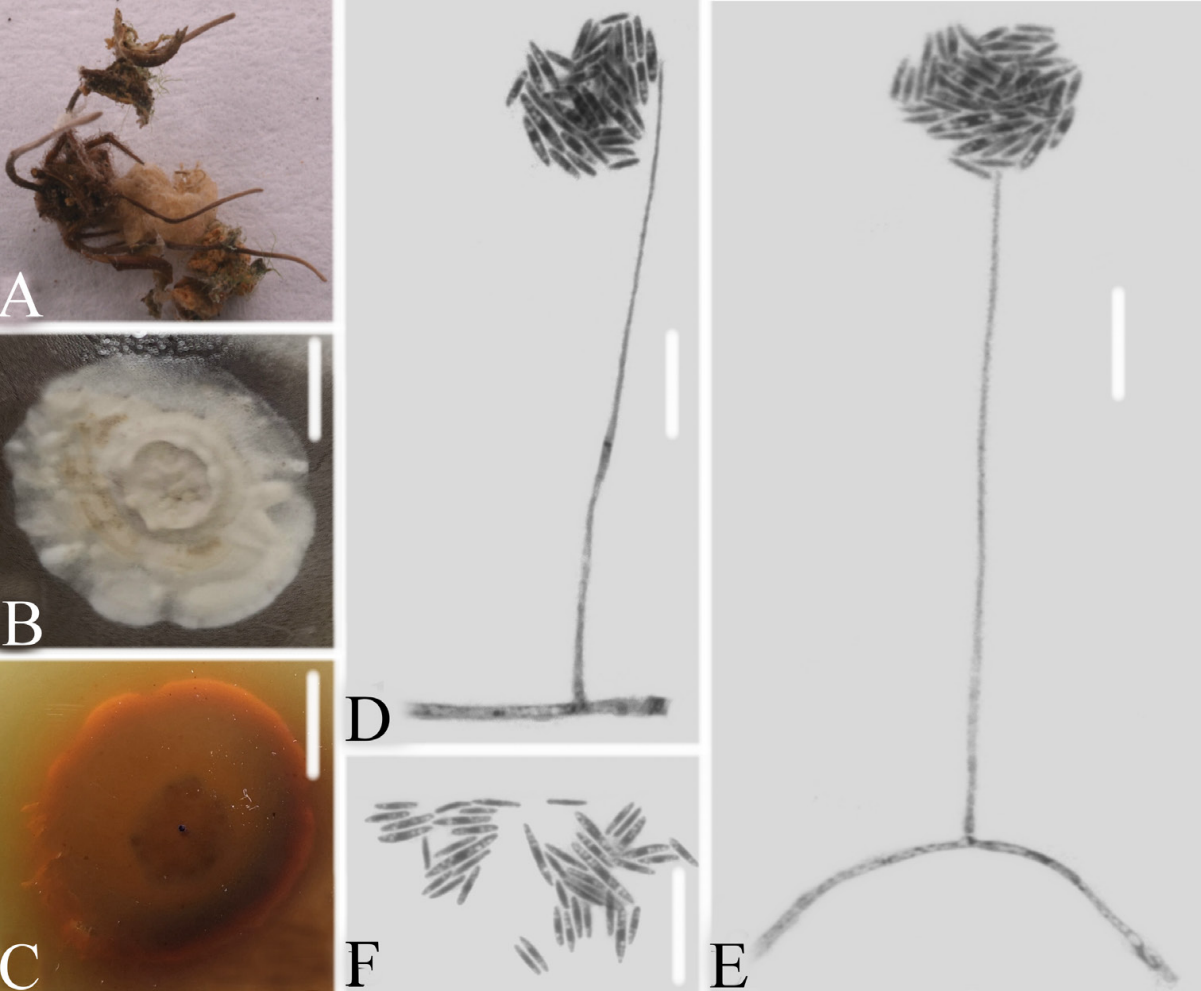
*Simplicillium formicidae***A** isolated substrate an infected ant (Hymenoptera) **B–C** culture plate, showing the front (**B**) and the reverse (**C**) of the colony, cultured on PDA medium **D, E** phialides solitary, conidia adhering globose slimy head and conidia **F** conidia. Scale bars: 10 mm (**B, C**), 10μm (**D, E, F**).

#### Description.

Colonies reaching 48–51 mm in diameter in 14 d on PDA; white; reverse pale white. Hyphae septate, hyaline, smooth-walled, 1.1–2.2 μm wide. Phialides arising from aerial hyphae, gradually tapering towards the apex, without basal septa, always solitary and rather long and narrow, 15.3–26.2 × 0.7–1.4 μm. Conidia adhering in globose slimy heads, ellipsoidal to fusiform, hyaline, smooth-walled, 1.6–2.4 × 1.4–1.7 μm. Octahedral crystals absent.

#### Host.

Carpenter worm (Lepidoptera)

#### Distribution.

Huaxi District, Guizhou Province, China

#### Remarks.

*Simplicillium lepidopterorum* was easily identified as belonging to *Simplicillium* because of its solitary phialides, conidia adhering in globose slimy heads, and lack of octahedral crystals. Comparing with the typical characteristics of 12 species (Table 2), *S. lepidopterorum* could easily distinguished from other species by having the phialides always solitary and rather long and narrow, 15.3–26.2 × 0.7–1.4 μm. Conidia ellipsoidal (1.6–2.4 × 1.4–1.7 μm), adhering in globose slimy heads, and in having the octahedral crystals absent. Based on ITS and LSU rDNA, *S. lepidopterorum* is phylogenetically close to *S. cicadellidae* and *S. formicidae*. However, *S. lepidopterorum* has ellipsoidal conidia, longer phialides (15.3–26.2 × 0.7–1.4 μm), and the reverse of colony was pale white.

##### Key

**Table:** 

1	Conidia in globose or subglobose heads	**2**
–	Conidia in chains or solitary	**11**
2	Macro- and microconidia present	**3**
–	Only one type of conidia present	**4**
3	Octahedral crystals present	***S. lamellicola***
–	Octahedral crystals absent	***S. coffeanum***
4	Octahedral crystals present	**5**
–	Octahedral crystals absent	***9***
5	Conidia cylindrical	***6***
–	Conidia subglobose or ellipsoidal	***7***
6	Chlamydospores present, conidia 4.2–6.5 × 1.2–2.0 μm	***S. aogashimaense***
–	Chlamydospores absent, conidia 3.0–4.5 (–5.0) × 1.0–2.0 μm	***S. cylindrosporum***
7	Conidia subglobose to ellipsoidal	**8**
–	Conidia oval or ellipsoidal to subcylindrical, 1.5–3.0 × 0.7–1.3 μm	***S. lanosoniveum***
8	Conidia subglobose to ellipsoidal, 2.3–4.0 (–4.5) × 1.5–3.3 μm	***S. subtropicum***
–	Conidia globose to subglobose, sometimes ellipsoidal, 2.5–3.5 × 1.8–2.5 (–2.8) μm	***S. minatense***
9	Conidia ellipsoidal	**10**
–	Conidia filiform to fusoid	***S. formicidae***
10	The reverse of colony pale white, phialide 12.9–18.3 × 0.8–1.1 μm	***S. cicadellidae***
–	The reverse of colony yellowish, phialide 15.3–26.2 × 0.7–1.4 μm	***S. lepidopterorum***
11	Denticles present in conidiogenous cell (phialide)	***S. sympodiophorum***
–	Denticles absent in conidiogenous cell (phialide)	**12**
12	Macro- and microconidia present	***S. calcicola***
–	Only one type of conidia present	**13**
13	Conidia ellipsoidal	**14**
–	Conidia fusoid to filiform, form zigzag chains	***S. filiforme***
14	Conidia in branched or unbranched chains, 3.5–5.0 × 1.0–1.5 μm	***S. chinense***
–	Conidia in short imbricate chains, 2.5–3.5 × 1.0–2.0 μm	***S. obclavatum***

## Discussion

Two types of the evolutionary correlation patterns between fungi and hosts are known, co-evolutionary patterns and the more frequent host jump events (Spatafora et al. 2007). The generation of host jumping is closely related to a common living environment (Vega et al. 2009). Nutritional sources are very important factors in determining whether a host has undergone a host jump. The nutritional model of Hypocreales fungi is from plants (including living plants and plant residues) to animals (especially insects), and finally to fungi. Plants and their residues were the initial sources of nutrition for the common ancestor of Hypocreaceae and Clavicipitaceae. The jumps from plants to animals and then to fungi indicate that the fungal nutrient requirements have changed with the environment (Spatafora et al. 2007). Prediction of the characteristics and evolutionary placement of any given member should be based on the correlation between molecular-phylogenetic genealogy and nutritional preferences (Spatafora et al. 2007; Vega et al. 2009). Additionally, host insect species are an important diagnostic feature in the identification of entomopathogenic fungi.

Among the 12 reported *Simplicillium* species, *S. aogashimaense* (soil), *S. calcicola* (calcareous rock), *S. chinense* (decaying wood), *S. cylindrosporum* (soil), *S. minatense* (soil), *S. obclavatum* (air), *S. subtropicum* (soil) and *S. sympodiophorum* (soil) were isolated from soil, marine water, rock, decaying wood and air (Zare and Gams 2001; Liu and Cai 2012; Nonaka et al. 2013; Liang et al. 2017). *Simplicillium filiforme* and *S. coffeanum* were isolated as endophytic fungi from plants (Crous et al. 2018; Gomes et al. 2018). *Simplicillium lamellicola* belongs to the hyperparasite fungi (Shin et al. 2017). *Simplicillium lanosoniveum* was reported as both an endophytic and hyperparasite fungi (Baiswar et al. 2014). It has been reported that *Simplicillium* is pathogenic to insects. Unfortunately, there are limited reports of insect-related *Simplicillium*.

The hosts of *Simplicillium cicadellidae* and *S. lepidopterorum* were larvae of Cicadidae and Lepidoptera, which feed through piercing-sucking and chewing. Moreover, *S. formicidae* was isolated from an infected ant. These three strains are likely to receive nutrients from plants (including living plants and plant residues) and animals (especially insects) based on the evolutionary pattern of Hypocreales. *Simplicillium cicadellidae*, *S. formicidae* and *S. lepidopterorum* represent three new species based on their nutritional preferences. To our knowledge, this is the first report of insect-associated *Simplicillium* species.

ITS and LSU have been widely used in the identification of *Simplicillium* (Liu and Cai 2012; Nonaka et al. 2013; Zhang et al. 2017; Sliva et al. 2018). In the present study, the combined dataset (ITS+LSU) was used to analysis of phylogenetic relationships among the new taxa and other *Simplicillium* species. Additionally, RPB1, RPB2 and TEF loci were added to analysis that the relationship among *Simplicillium* and its allies. The new species clustered with other *Simplicillium* species in a clade (Fig. 1), and this was consistent with morphological characteristics based identification. Six strains were clustered into three subclades (Fig. 2) and were distinctly different from other reported *Simplicillium* spp. Additionally, three species, *S. chinense*, *S. coffeanum* and *S. filiforme* were clustered in a subclade, and these species were associated with plants. This may be because of their nutritional preferences. Therefore, *S. cicadellidae*, *S. formicidae* and *S. lepidopterorum* are based on morphological characteristics, ecological characteristics and a phylogenetic analysis.

## Supplementary Material

XML Treatment for Simplicillium cicadellidae

XML Treatment for Simplicillium formicidae

XML Treatment for Simplicillium lepidopterorum
